# Congenital syndactyly: a retrospective study of 18 cases at the Department of Orthopaedic Surgery and Traumatology of the Habib Bourguiba University Hospital, Sfax, Tunisia

**DOI:** 10.11604/pamj.2022.41.30.30253

**Published:** 2022-01-12

**Authors:** Ahmed Racem Guidara, Sahnoun Nizar, Tarek Bardaa, Moez Trigui, Sana Kmiha, Kamel Ayadi, Hassib Keskes

**Affiliations:** 1 Department of Orthopaedic Surgery and Traumatology, Habib Bourguiba University Hospital, Sfax, Tunisia,; 2 Pediatrics Department, Hedi Chaker University Hospital, Sfax, Tunisia

**Keywords:** Congenital syndactylies, flap, skin graft, polymalformative syndromes

## Abstract

Congenital syndactylies are frequent congenital malformations of the hand. They can be an isolated finding or they can be found in association with other polymalformative syndromes. Several surgical techniques used to treat them have been described in the literature. The most used is the dorsal commissural omega-flap technique. We here report a study of 18 patients with congenital syndactyly, with multiple involvement in several cases, whose data were collected at the Department of Orthopedics and Traumatology of Sfax (Tunisia). All patients were operated using the dorsal commissural omega-flap technique. We operated 42 commissures in 18 patients. The average age of patients was 7 years. Only 3 patients had syndromic forms. Six of these patients were operated in two stages. For scar quality, mean OSAS score was 11.47 (11.35 for simple types and 12 for complex types). All patients with complex types had long-term complications (100%). Six patients with simple types out of 14 had complications (42.85%). The management of congenital syndactylies is surgical. It is important to provide parents with accurate information on the essential role of follow-up appointments in order to avoid complications in the short and the long term.

## Introduction

Syndactylies are among the most common congenital malformations of the hand. They are manifested by the joining and fusion between two or more fingers. They can be isolated or integrated into polymalformative syndromes of which we mainly mention Apert syndrome, Poland's syndrome and amniotic fluid syndrome. The management of these deformations is surgical, based essentially on the realization of a flap associated or not with a skin graft. In fact, several techniques are available for this operation. The most used is the commissural dorsal omega flap. The aim of this work is to report the different types of congenital syndactylies and the most frequent syndromic forms, the application of commissural dorsal flap in omega surgical technique and evaluate its results.

## Methods

### Study design and sampling

A retrospective study was carried out in the orthopedic department of Habib Bourguiba University hospital in Sfax - Tunisia. The study concerned the patients operated for a congenital syndactyly from 2000 until 2016.

**Figure 1:**
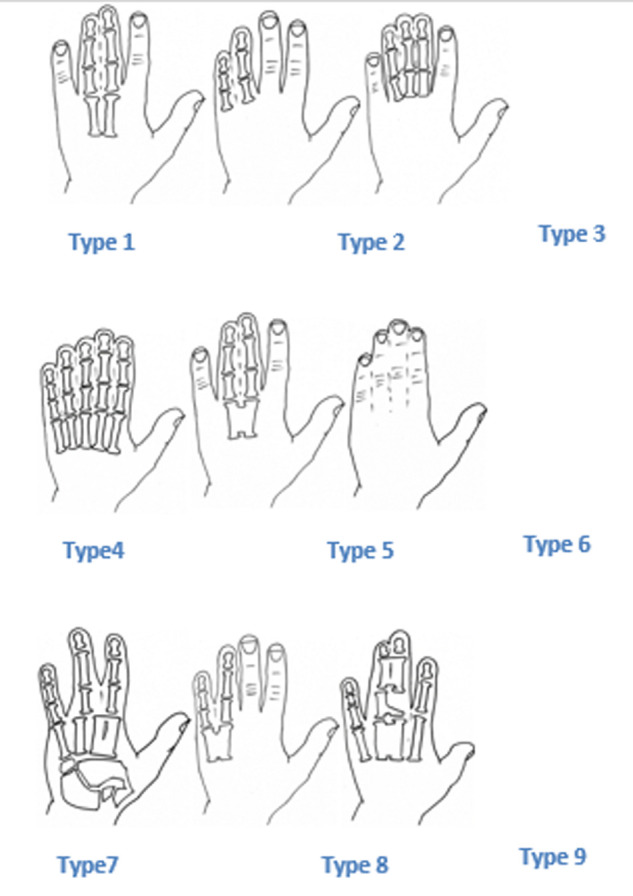
temtamy and McKusick classification [1]

**Figure 2:**
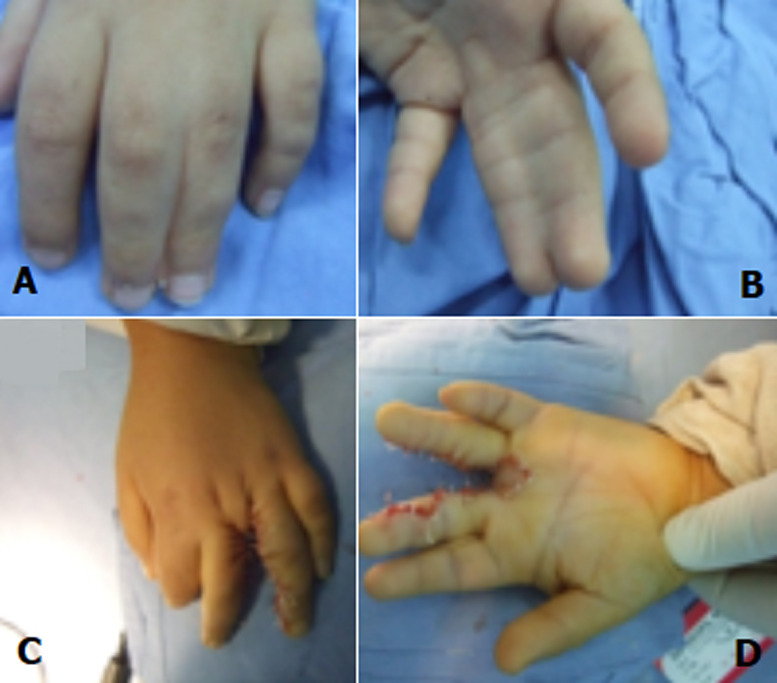
anatomic classification of congenital syndactylies; simple incomplete (A); simple complete (B), complex (C), complicated (D)

Patients undergoing congenital syndactyly of the hand, whether simple or complex, syndromic or not and those who were treated surgically with commissural dorsal omega flap, with or without skin graft were all included in the study. Patients diagnosed but not treated, those with unusable or missing medical data and those with insufficient follow-up of at least 6 months were excluded. We reviewed files in order to recognize and classify different types and clinical forms of syndactylies that may be associated with poly-malformative syndromes. Sporadic non-syndromic forms were classified using the Temtamy and Mckusik classification [1] (Figure 1). The operation is performed under general anesthesia. A pneumatic tourniquet is placed at the operated limb´s root. The tourniquet size must be adapted. The omega flap is marked on the dorsal surface of the finger. Its base corresponds to the metacarpal heads and its vertex reaches the proximal interphalangeal joint level. On the palmar surface, incisions are made to form an anchor that starts at the proximal interphalangeal joint and descends few millimeters below the level of the desired web. The lateral anchor´s branches are joined at the base of two adjacent fingers. Thus, Fingers are separated beyond the proximal interphalangeal joint by symmetrical Z- incisions, both on the dorsal and the palmar side. The incision starts from the commissural flap and continues at the ends of the fingers. On the dorsal side, it starts from the proximal interphalangeal joint of a finger, through the middle of the second phalanx of the other finger and then arrives on the distal interphalangeal joint of the first finger and finally on the third phalanx of the second. In the palmar face, incisions should correspond to the dorsal incisions. Incisions is not allowed to exceed the median line of each finger [2] (Figure 2). The dissection of the dorsal flap and the separation of fingers continues proximally to the branching area of the common neurovascular bundle. The tip of the dorsal flap is then sutured with a resorbable 4/0 thread to its palmar recipient site corresponding to the top of the anchor. The two palmar lateral flaps are sutured on the lateral side of the web. In case of cutaneous substance loss, the denuded areas on the lateral faces are covered with small total skin grafts taken from the crease of the groin or the palmar side of the wrist. In acro-syndactyly, distal bone fusion is separated first by simple division [3]. The operated area is covered with fat and wet dressing. In any case, dry dressing is never opted for. The dressing is bulky including the whole hand, ideally reinforced by an elastoplast, well attached to the forearm. Hospitalization lasted 24 hours on average. The first dressing change was made after 7 to 10 days, then weekly until healing. Patients were then seen again after 1 to 3 months, then regularly every year until skeletal maturation.

Eighteen patients among the 42 operated commissures were summoned to the department to evaluate the surgical technique and to check for short-term and long-term postoperative complications. Our judgment was based on OSAS (Observer Scare Assessment Scale) score (Table 1) and Whitey´s score (Table 2). We also looked for other abnormalities such as clinodactyly, digital flexion, commissural retraction, an anomaly of the fingernailor a disorder of digital mobility.

**Table 1:** observer scare assessment scale (OSAS)

Normal Skin	1	2	3	4	5	6	7	8	9	10
Vascularization	1	2	3	4	5	6	7	8	9	10
Pigmentation	1	2	3	4	5	6	7	8	9	10
Thickness	1	2	3	4	5	6	7	8	9	10
Relief	1	2	3	4	5	6	7	8	9	10
Pliability	1	2	3	4	5	6	7	8	9	10
Total Score										

**Table 2:** commissure evaluation according to Whitey

Whitey score	Commissure appearance
0	Normal appearance of the commissure
1	Opening reduction without web creep
2	1/3 of P1 web creep
3	2/3 of P1 web creep
4	All P1 web creep

### Statistical analysis of the data

Qualitative data were described using number and percent. Quantitative data were described using mean and standard deviation.

## Results

### Socio-demographic characteristics

We operated 42 commissures in 18 patients. The sex ratio was 1.57.

### Descriptive study

**Figure 3:**
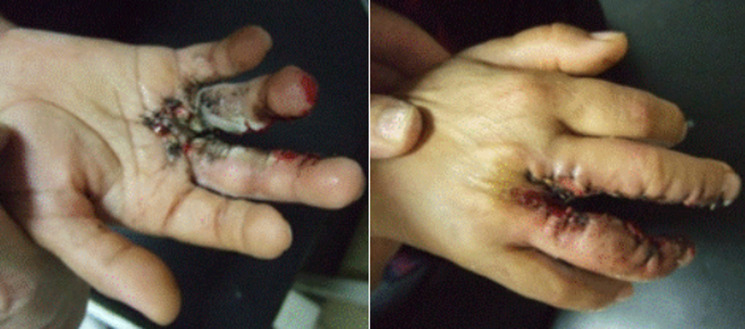
post-operative infection

Congenital syndactyly was unilateral in 61.11% of cases. Only 3 patients presented a syndromic form: Apert syndrome. The remainder (15 patients) were sporadic forms, including Temtamy and McKusick classification type 1 in 12 cases (80% of cases), and 3 cases type 3 (20% of cases). Associated non-labeled malformations were found in 7 patients (38.88%): 2 patients with middle finger agenesis, 1 patient with club foot, 3 patients with congenital syndactyly of the feet and 1 patient with dysmorphic syndrome, interventricular communication and ptosis. All our patients were operated using the dorsal commissural omega flap technique, six among which were operated in two stages. The average time between the first and the second operation was 15 months. Three (3) patients received a total skin graft. The sample was taken from the anterior side of the wrist in 2 cases. Note that there were no intraoperative complications. The average follow-up lasted 50 months. Short-term complications were recoreded in 6 patients. Infection occurred in 4 cases (Figure 3). These patients received antibiotic therapy and operative wound cleaning with aseptic compliance. Fleshy bud was found in 2 cases whose dressing was poorly done with contact between the two operated fingers. Palmar bridle was recorded in only one case. A release of this bridle was performed with good result. For the scar quality, the mean OSAS score was 11.47. It was 11.35 for simple shapes and 12 for complex shapes.

Long-term complications (Table 3) were also recorded. Flexum in 6 fingers, clinodactyly in 7 fingers, commissural retraction in 1 case, nail dystrophy in 4 cases and surgical revision in 1 case. All complex forms presented long-term complications (100%). Six simple forms out of 14 presented complications (42.85%). These complications were favored by the retractions of skin scars or ligaments Table 4, Table 5 summarize all characteristics and long term complications found in our series.

**Table 3:** distribution of long-term complications by type of syndactyly

	Simple partial	Simple complete	Complex	TOTAL
Flexum	2	0	4	6
Clinodactyly	1	1	5	7
Commissural retraction	0	0	1	1
Nail dystrophy	1	1	2	4
Surgical revision	0	0	1	1

**Table 4:** recapitulation of clinical features of syndactylies and classifications

Patient	Age (years)	Sex	Affected side(s)	Affected commissures	Hand(s) radiographs	Syndromic / Non- syndromic/Not labeled associated malformation	Temtamy and McKusick Classification	Anatomic Classification
1	8	F	Bilateral	4	Normal	Non-syndromic	Type 3	Simple complete
2	27	F	Left	4	Normal	Non-syndromic	Type 1	Simple partial
3	6	F	Bilateral	3 + 4	Bone synostosis	Apert syndrome	-	Complex
4	6	M	Left	4	Normal	Non-syndromic	Type 1	Simple partial
5	12	M	Bilateral	3 + 4	Normal	Non-syndromic	Type 1	Simple complete
6	19	F	Right	3	Normal	Non-syndromic	Type 1	Simple complete
7	8	M	Right	3	Normal	Dysmorphic syndrome +Interventricular communication + Ptosis	Type 1	Simple partial
8	4	M	Bilateral	3 + 4	Bone synostosis	Apert syndrome + Left soft clubfoot	-	Complex
9	7	M	Left	3	Normal	Non-syndromic	Type 1	Simple complete
10	6	M	Left	3	Normal	Toes syndactyly	Type 1	Simple partial
11	7	M	Bilateral	3 + 4	Normal	Non-syndromic	Type 3	Simple complete
12	31	M	Left	3 + 4	Normal	Non-syndromic	Type 1	Simple complete
13	5	F	Bilateral	4	Normal	Non-syndromic	Type 3	Simple complete
14	15	F	Left	3	Normal	Non-syndromic	Type 1	Simple complete
15	15	F	Bilateral	3	Normal	Toes syndactyly	Type 1	Simple partial
16	24	M	Bilateral	2+ 3 + 4	Normal	Toes syndactyly	Type 1	Simple complete
17	5	M	Right	All	Bone synostosis	Apert syndrome + Agenesis of a central ray	-	Complex
18	18	M	Right	1 + 4	Normal	Agenesis of a central ray	Type 1	Simple complete

**Table 5:** recapitulation of follow-up andlong-term complications

Patients	Follow-up (years)	OSAS score	Whitey score	Mobility	Clinodactyly	Retraction	Flexum	Nail
1	4	12	0	Normal	No	No	Yes	Good condition
2	0,5	6	0	Normal	Yes	No	No	Good condition
3	5	11	2	Normal	Yes	Yes	No	Good condition
4	4	11	0	Normal	No	No	No	Good condition
5	2	11	0	Normal	Yes	No	No	Dystrophic
6	1	7	0	Normal	No	No	No	Good condition
7	7	6	0	Normal	No	No	No	Good condition
8	3	13	1	Normal	Yes	No	Yes	Dystrophic
9	1	15	0	Normal	No	No	No	Good condition
10	1	11	0	Normal	No	No	No	Dystrophic
11	5	9	0	Normal	No	No	Yes	Good condition
12	4	19	0	Normal	No	No	No	Good condition
13	3	8	0	Normal	Yes	No	No	Good condition
14	10	11	0	Normal	No	No	No	Good condition
15	10	12	0	Normal	No	No	No	Good condition
16	10	21	2	Normal	No	No	No	Good condition
17	0,5	12	0	Abnormal	No	No	No	Dystrophic
18	Lost patient							

## Discussion

In simple syndactyly, only the skin is affected. While in complex syndactyly, there are bone abnormalities, mainly bone fusion, and other abnormalities such as supernumerary rays, absence of radius, phalanx delta and brachyphalangia [4]. Tendon abnormalities may also be present. These include insertion anomaly and absent or supernumerary tendons. Finally, vascular abnormalities can be a distal division of the common digital artery, single artery, or a complete absence of artery in the concerned commissure [5]. There are 4 types of congenital syndactylies. They may be simple and partial, in two types: type B corresponds to a joining until the second phalanx. Type C corresponds to a joining until the first phalanx. They may be simple and complete, also known by type A. They can also be complex or complicated. Our study showed a clear predominance of simple and complete syndactylies (59.52%) compared to partial (14.28%) and complex forms (26.19%).

These results are different from those found by Mandrano Filbo [6] in 2013. They found that the most frequent form was the complex syndactyly (41%) followed by the complete simple form (38.35%) and finally by the simple partial form (20%). There are syndromic forms and non-syndromic (sporadic) forms. The most common syndrome is Apert Syndrome: Described by Eugène Apert in 1906, Apert syndrome is an acro-cephalo-syndactyly. It is an autosomal dominant transmission disorder with sporadic mutations. Its incidence is in about 1/45000 births. It associates a facio-cranio-synostosis, exorbitism, a retrusion of the middle third of the face and a characteristic lesion of the hands and feet, bilateral and always symmetrical [7-10]. The second syndrome is Poland Syndrome which is characterized by a thoracic anomaly associated with abnormalities of the ipsilateral upper limb. The incidence is estimated at 1 per 30,000 births. The right side is affected in 75% of cases [11]. The hand is the most affected. The main malformation is brachy-mesophalangia resulting in brachydactyly. A syndactyly is usually associated. Other anomalies may also be associated such as polydactyly, camptodactyly and delta phalanx [12-16].

The main thoracic anomaly is the agenesis of the stern coral chiefs of the pectoralis major, which is clinically manifested as infra-clavicular depression and absence of anterior axillary abutment. There have been no cases of this syndrome in our series. It is a rare congenital syndrome resulting from the trapping of the fetus in strands of amniotic tissue, which causes a series of deletions and deformities. It was estimated that one per 10,000 births had this syndrome. This syndrome is underestimated and its presentation is so variable that there are no two exactly similar cases [17]. Amniotic flank disease can be associated with brachy-syndactyly and intrauterine amputations. Flanges can be responsible for compressing noble structures. Long fingers are the most affected in the hand, unlike the foot, where the hallux is most often affected [17-19].

Pfeiffer syndrome is a rare syndrome with autosomal dominant inheritance. It results from a mutation in the fibroblast growth factor receptor gene (FGFR1 or FGFR2) [20]. This syndrome affects about 1 person per 100,000. It combines craniosynostosis, enlarged and deviated thumbs and toes with partial syndactyly of hands and feet. Other less common abnormalities may also be seen, such as hydrocephalus, ocular exophthalmia, joint and shoulder ankylosis, visceral abnormalities, and developmental delay [20-22]. The antenatal diagnosis of Pfeiffer syndrome can be made by prenatal ultrasound findings such as craniosynostosis, exophthalmos, and wide thumb.

Genetic testing is important to confirm the diagnosis. Treatment includes a craniotomy for craniosynostosis. Surgery can be performed to reduce exophthalmia and maxillary hypoplasia especially in severe cases [23]. Surgery aims to reproduce a normal interdigital commissure appearance. The three-main commissural dorsal flaps used are the rectangular flap, the Gilbert omega flap (the used method in our practice) and the Glicenstein T flap [3,24]. According to Mallet [25], the omega commissural dorsal flap technique has a lower risk of web creep compared to the T flap technique. Indeed, it allows the commissure reconstruction with a natural form and avoids the use of skin grafting on the lateral faces. This also makes it possible to limit the number of retractions and the occurrence of secondary deformities (clinodactyly, flessum...).

As a result, all our patients were operated on by omega flap. Only 3 patients required total skin grafting which does not contradict the literature. However, the disadvantage of these flaps is the risk of dermal cutaneous tissue loss requiring, in some cases, a skin graft. As a result, new techniques have emerged to address this problem: Z-incision, palmar incision, inter-metacarpal islet flaps and retrograde inter-metacarpal flaps [26-29].

Our results in terms of postoperative complications relate to those of D´Arcangelo (3) (1996) who found that only 9 cases required surgical revision in his series of 122 syndactyly (50 patients) operated by dorsal omega flap. Eight cases developed a web creep. Only 3 cases of commissural retraction were found. Complete forms and complex forms were the most likely to cause postoperative complications.

### Limitations

We used convenience sampling technique; hence the generalization of results would not be possible because we presented.

## Conclusion

Syndactyly is one of the most common congenital malformations of the hand. It can be isolated (sporadic form) or associated to poly-malformative syndromes. The management is surgical, based essentially on the realization of flaps. A skin graft can be associated but is now being abandoned because of its complications. A meticulous surgical technique, a sufficient dressing that respects the vascularization of flaps, as well as informing parents by the need of a follow-up until adolescence, are vital conditions to avoid the occurrence of complications in the short and the long terms.

### What is known about this topic

- Syndactylies are rare;
- Several surgical techniques have been described;
- The most used is the commissural dorsal omega flap.

### What this study adds

- A skin graft can be associated but is now being abandoned because of its complications;
- A meticulous surgical technique, a sufficient dressing that respects the vascularization of flaps, are vital conditions to avoid the occurrence of complications in the short terms;
- Informing parents by the need of a follow-up until adolescence, are vital conditions to avoid the occurrence of complications at the long terms.
